# From discovery to outbreak: the genetic evolution of the emerging Zika virus

**DOI:** 10.1038/emi.2016.109

**Published:** 2016-10-26

**Authors:** Hong Liu, Liang Shen, Xiao-Lin Zhang, Xiao-Long Li, Guo-Dong Liang, Hong-Fang Ji

**Affiliations:** 1 Shandong Provincial Research Center for Bioinformatic Engineering and Technique, School of Life Sciences, Shandong University of Technology, Zibo 255049, Shandong Province, China; 2 State Key Laboratory of Infectious Disease Prevention and Control, National Institute for Viral Disease Control and Prevention, Chinese Center for Disease Control and Prevention, Beijing 102206, China; 3 Collaborative Innovation Center for Diagnosis and Treatment of Infectious Diseases, Hangzhou 310058, Zhejiang Province, China

**Dear Editor**,

Zika virus (ZIKV) was initially isolated from a rhesus monkey in Uganda in 1947.^1^ Thereafter, ZIKV was identified in both mosquitoes and humans, and mosquitoes are the transmitting vector of ZIKV.^2^ Historically, only a few infectious cases were reported worldwide before the 21st century, but in 2007, a large ZIKV epidemic occurred on Yap island.^3^ Another outbreak is currently spreading throughout South America, resulting in a large number of cases of microcephaly,^4^ Guillain–Barre syndrome^5^ and viral meningoencephalitis.^6^ Unlike other arboviruses, ZIKV can be transmitted through sexual contact. Thus, the characteristics of ZIKV infection are more complex and diverse than those of any other arboviral disease. For these reasons, the World Health Organization (WHO) has declared the present ZIKV outbreak as an international public health issue.^7^

A phylogenetic analysis of ZIKV sequences identified two major lineages: the African and Asian lineages.^8, 9^ Asian strains of ZIKV are responsible for the outbreaks occurring in South America since 2015. Since the beginning of the 21st century, a significant increase in the number of ZIKV cases has been reported.^2^ Although ZIKV has been circulating in nature for almost 70 years, the reason for the sudden increase in the virulence and transmission capacity of the virus is unknown. The underlying mechanisms of ZIKV molecular evolution are urgently in need of clarification. In this study, we used Bayesian methods in a phylogenetic analysis of whole-genome sequences from the currently circulating ZIKV strains to determine the genetic evolutionary trends of ZIKV and provide some clues for ZIKV prevention and control.

The complete genome sequences from a total of 55 ZIKV isolates were obtained from the GenBank database (as of 1 May 2016). These stains were isolated from a variety of hosts: monkeys (*n*=2), mosquitoes (*n*=14) and humans (*n*=39). The isolation dates range from 1947 to 2016, and the locations of isolation include all regions in which ZIKV cases have been identified (Figure 1A). Bayesian time-scaled phylogenetic analysis, analysis of the molecular evolutionary rates of the entire ZIKV population and of each ZIKV genotype, and the time to the most recent common ancestor (TMRCA) of ZIKV were estimated comprehensively using the BEAST software package.^10^ The GTR+I+G substitution model was selected as the best-fit nucleotide substitution model by MrModelTest. The relaxed log clock model and Bayesian skyline plot demographics were chosen as the best-fit parameters. The analysis was run through 50 000 000 generations to ensure sufficient mixing. Finally, the maximum clade credibility (MCC) tree was built using TreeAnnotator with 10% burn-in (http://beast.bio.ed.ac.uk/). The MCC tree based on the whole genome of each ZIKV strain is shown in Figure 1A. The posterior probability values for the nodes of each lineage were >0.8, indicating their robustness. The most recent common ancestor for all ZIKV genotypes was estimated to have arisen ~155 years ago (95% highest posterior density (HPD): 85.01–261.13), and this common ancestor subsequently evolved into two main populations: the African and Asian lineages. The TMRCA for the Africa lineage was estimated at 127 years ago (95% HPD: 84.92–180.87), while that of the Asian lineage was estimated at 68 years ago (95% HPD: 50.85–97.22). The African lineage is divided into two independent evolutionary groups: African sublineages I and II. The TMRCA for African sublineage I, which comprises two isolates from monkeys and nine from mosquitoes, was estimated at 101 years ago (95% HPD: 75.45–133.77). The TMRCA for African sublineage II, which includes two strains isolated from patients in 1968, was estimated at 75 years ago (95% HPD: 54.90–98.55). The Asian lineage could also be divided into two groups based on the host species: Asian sublineages I and II. For Asian sublineage I, the single isolate from a mosquito (P6-740/Malaysia/1966) forms an independent evolutionary branch and is the oldest member of the Asian lineage. All of the human strains isolated from 2007 to 2016 cluster together to form Asian sublineage II. This sublineage emerged 18 years ago (95% HPD: 10.74–28.50) and is the youngest lineage within the entire ZIKV population. All of the strains isolated from the epidemic in 2015 to 2016, together with the Haiti225 (Haiti, 2014) isolate and the H/PF2013 (French Polynesia, 2013) isolate, exhibited a close relationship and formed a group. Moreover, the H/PF2013 strain is located close to the root of the phylogenetic group, indicating that it is likely the ancestor of the other strains in this population. Furthermore, the TMRCA for this group was estimated at 4.31 years ago (95% HPD: 3.09–5.88), suggesting that the ancestor of this sublineage was circulating in 2012.

The TMRCA for all ZIKV strains was estimated to be 155 years ago in our current study, while the TMRCA for all Japanese encephalitis virus strains^11^ was estimated to diverge approximately 1695 years ago and those for West Nile virus^12^ and yellow fever virus^13^ at approximately 200 years ago and 306 years ago, respectively. Thus, ZIKV can be considered an ‘emerging' arbovirus. Substitution rate is an important parameter for understanding viral evolution. In this study, the mean nucleotide substitution rate for the entire ZIKV population was estimated at 6.091 × 10^−4^ substitutions per site per year (s/s/y) (95% HPD, 3.44 × 10^−4^ to 8.79 × 10^−4^). Furthermore, the estimated substitution rate for the African lineage was 2.689 × 10^−4^ s/s/y (95% HPD, 6.241 × 10^−6^ to 6.306 × 10^−4^), while it was 1.0233 × 10^−3^ s/s/y (95% HPD, 8.2 × 10^−4^ to 1.2 × 10^−3^) for the Asian lineage. Surprisingly, the substitution rate of the currently circulating Asian lineage of ZIKV is considerably higher than those of the African lineage (pre-pandemic ZIKV population) and other mosquito-borne flaviviruses, all of which have substitution rates at 10^−4^ s/s/y. Considering that a higher substitution rate often enhances viral adaptability and competitiveness, as well as pathogenicity,^14^ this rate could be a contributing factor in the sudden outbreak of ZIKV caused by strains of the Asian lineage.

According to our results, since the marked increase in cases of ZIKV infection in the 21st century, obvious differences have arisen between the African and Asian lineages with respect to genetic diversity. As shown in Figure 1B, the genetic diversity of the African lineage has remained relatively stable over time, while the Asian lineage (Figure 1C) underwent more active changes and maintained a relatively higher level of diversity than that of the 2013 ZIKV outbreak in French Polynesia.

Our work provides further insight regarding the molecular evolutionary differences between the African and Asian lineages of ZIKV. The Asian lineage, which is the youngest genotype, has a fast rate of evolution and a marked increase in genetic diversity, which provides this lineage with a selective advantage. Notably, a recent report by the WHO on the spread of the Asian lineage of ZIKV beyond South America to the country of Cape Verde in West Africa^7^ indicates the potential for ZIKV to spread to non-endemic areas. The ZIKV infections that occurred in 2015 were mainly distributed in tropical countries such as Brazil in South America, but countries in the northern hemisphere were not conductive for mosquito breeding in the winter. However, with the northern hemisphere gradually entering into the summer season, with increasing temperature, precipitation and mosquito density, ZIKV will likely spread northward. Therefore, enhanced detection and surveillance of ZIKV in the northern hemisphere is highly recommended despite the current pandemic being restricted to the southern hemisphere.

## Figures and Tables

**Figure 1:**
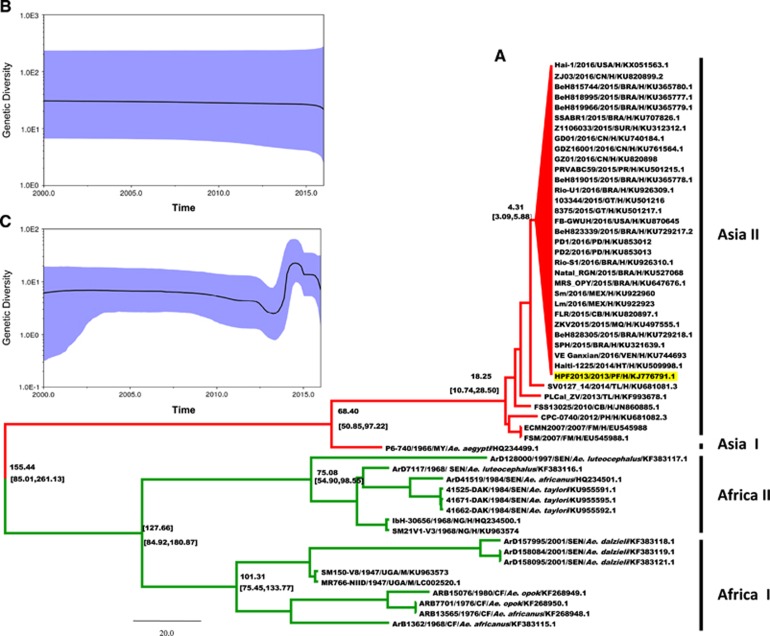
Maximum clade credibility tree and Bayesian skyline plots for African and Asian lineages of ZIKV. (**A**) Maximum clade credibility tree for 55 whole-genome sequences of ZIKV obtained from isolates collected in 1947 to 2016. Consistent with previous studies, the tree is divided into two distinct lineages: the African lineage (green) and the Asian lineage (red). Estimated TMRCAs of these lineages (with their 95% HPD values in parentheses) are shown. The current pandemic ZIKV group of Asian lineage is shown as a red triangle, and strain H/PF2013 is highlighted in yellow. Viral nomenclature is as follows: virus strain/year of isolation/country/origin/accession number. BRA, Brazil; CB, Cambodia; CN, China; FM, Micronesia; GF, Central African Republic; GT, Guatemala; H, Human; HT, Haiti; M, Monkey; MEX, Mexico; MQ, Martinique; MY, Malaysia; NG, Nigeria; PD, Padua; PF, French Polynesia; PH, Philippines; PR, Puerto Rico; SEN, Senegal; SUR, Suriname; TL, Thailand; UGA, Uganda; VEN, Venezuela. (**B**, **C**) Bayesian skyline plots for African and Asian lineages, separately, since the beginning of the 21st century. The horizontal and vertical axes represent time and genetic diversity, respectively. The highlighted areas correspond to 95% HPD intervals.
